# In Silico Screening of Plant-Derived Termiticidal Compounds Targeting Cytochrome P450 in *Coptotermes* spp. (Blattodea: Rhinotermitidae) for Sustainable Termite Management

**DOI:** 10.3390/plants15040581

**Published:** 2026-02-12

**Authors:** Deepak Kumar Mahanta, Tanmaya Kumar Bhoi, Sumit Jangra

**Affiliations:** 1 Forest Protection Division, Indian Council of Forestry Research and Education-Forest Research Institute (ICFRE-FRI), Dehradun 248006, Uttarakhand, India; 2 Forest Protection Division, ICFRE-Arid Forest Research Institute (ICFRE-AFRI), Jodhpur 342005, Rajasthan, India; tkbhoi@icfre.org; 3 UF/IFAS Tropical Research and Education Center, Homestead, FL 33031, USA

**Keywords:** botanical insecticides, cytochrome P450 inhibition, termite detoxification mechanisms, in silico screening, environmental risk assessment, sustainable termite management

## Abstract

Termites of the genus *Coptotermes* are among the most destructive structural pests worldwide, owing to their efficient lignocellulose degradation and metabolic adaptability mediated in part by cytochrome P450 enzymes. Although numerous botanical compounds have been reported to exhibit termiticidal activity, mechanistic in silico studies targeting detoxification-related enzymes in *Coptotermes*, particularly cytochrome P450, remain limited. In this study, twenty-eight plant-derived bioactive compounds were evaluated using an integrated in silico framework comprising insecticide likeness screening, molecular docking, toxicity prediction, environmental fate assessment, and molecular dynamics simulation. Homology modeling enabled structural characterization of cytochrome P450 from *C. formosanus*, and subsequent screening identified 27 compounds with favorable physicochemical and ADMET properties. Molecular docking analysis highlighted Glyceollin, Cnicin, Biochanin A, Ferruginol, and ent-kaur-16-en-19-oic acid as strong binders, exhibiting stable interactions with conserved active-site residues. Toxicological and ecological assessments indicated generally low predicted risk to mammals, birds, and pollinators, while identifying potential sensitivity in aquatic organisms, emphasizing the need for controlled application. Molecular dynamics simulations further supported the stabilizing effect of Glyceollin on cytochrome P450 under simulated conditions. Overall, the study provides mechanistic insight into botanical inhibition of cytochrome P450 in *Coptotermes* and identifies promising candidate compounds for further experimental validation in sustainable termite management strategies.

## 1. Introduction

Termites represent one of the most evolutionarily successful insect groups, having developed complex eusocial organization and highly efficient systems for resource utilization [1]. Among them, species belonging to the genus *Coptotermes* are regarded as some of the most destructive subterranean termites worldwide due to their exceptional capacity for lignocellulose degradation, supported by symbiotic gut microorganisms and host metabolic enzymes [2]. Their broad feeding range, large colony size, and rapid wood consumption result in extensive damage to wooden structures, agricultural commodities, and living trees [3,4]. Subterranean termites, particularly *Coptotermes* spp., are widely distributed across tropical and subtropical regions and are responsible for substantial economic losses, estimated at approximately USD 32 billion annually [5,6]. Their invasive nature and human-mediated dispersal have further accelerated their global spread, making them a persistent and challenging pest in many regions [4,7,8,9,10,11].

Current termite management strategies rely largely on chemical termiticides, the effectiveness of which depends on factors such as termite biology, infestation intensity, soil characteristics, and application practices [12,13]. However, prolonged use of synthetic chemicals has raised serious concerns related to groundwater contamination, non-target toxicity, bioaccumulation, and the emergence of resistance [14]. Although non-chemical approaches, including physical, cultural, and biological methods, have been explored [15,16,17,18], their limited persistence and inconsistent field performance restrict widespread adoption. Consequently, botanical insecticides have gained attention as sustainable alternatives due to their biodegradability, structural diversity, and multi-target modes of action [19,20,21]. Despite extensive screening of plant-derived compounds, only a small proportion have progressed toward practical termite control applications.

Cytochrome P450 enzymes constitute a critical detoxification system in insects, enabling the metabolism of plant allelochemicals, environmental toxins, and synthetic insecticides. In termites, cytochrome P450s play a vital role in sustaining survival under continuous exposure to lignocellulosic by-products and xenobiotics encountered during wood feeding. Enhanced P450-mediated detoxification has also been implicated in metabolic tolerance and resistance to control agents. Therefore, inhibition of cytochrome P450 represents a biologically rational strategy for termite management, as disruption of this pathway may impair detoxification capacity, compromise physiological homeostasis, and increase susceptibility to bioactive compounds.

The aim of this study was to identify plant-derived bioactive compounds capable of inhibiting cytochrome P450 in *C. formosanus* using an integrated in silico framework. The key research questions addressed were: (i) which plant-based compounds exhibit strong binding affinity toward termite cytochrome P450, (ii) whether these compounds possess favorable insecticide likeness and toxicity profiles, and (iii) how ligand binding influences the structural stability of the target protein. The study was guided by the hypothesis that selective inhibition of cytochrome P450 can disrupt termite detoxification mechanisms and represent an effective molecular target for botanical termiticides. Accordingly, the specific objectives were to perform molecular docking, ADMET (Absorption, Distribution, Metabolism, Excretion, and Toxicity) and ecological toxicity assessment, environmental fate analysis, and molecular dynamics simulation to identify promising and environmentally cautious candidates for sustainable termite management.

## 2. Materials and Methods

### 2.1. Retrieval of Ligands

A total of 28 plant-derived bioactive compounds with reported termiticidal or allelopathic activity were selected from published literature [22,23,24,25,26,27,28,29,30,31,32,33]. The three-dimensional (3D) structures of the ligands were retrieved from the PubChem database (https://pubchem.ncbi.nlm.nih.gov/, accessed on 1 November 2025) [34] in SDF format using their Compound IDs. Essential chemical information (IUPAC name, molecular formula, molecular weight, and optimized 3D geometry) was obtained directly from PubChem. The complete list of compounds, plant sources, and references is provided in the corresponding table.

### 2.2. Protein Retrieval, Homology Modelling and Characterisation

The amino acid sequence of cytochrome P450 from *C. formosanus* (NCBI Accession No. AFH78148.1) was retrieved from the NCBI Protein database (https://www.ncbi.nlm.nih.gov/, accessed on 15 November 2025) [35], as no experimentally resolved structure is available. Homology modelling was performed using the SWISS-MODEL server (https://swissmodel.expasy.org/, accessed on 30 November 2025) [36]. Structural quality was assessed using Ramachandran plot analysis (PROCHECK) and ProSA-web Z-score evaluation [37,38]. Physicochemical properties were analysed using ProtParam (https://web.expasy.org/protparam/, accessed on 30 November 2025) [39]. The inherent limitations of homology modelling, particularly uncertainties in flexible regions and active-site geometry, were considered during docking and result interpretation.

### 2.3. Secondary Structure and Subcellular Localisation Analysis

Secondary structure elements were predicted using NetSurfP-3.0 [40]. Subcellular localisation and transmembrane topology were assessed using TMHMM v2.0 and DeepLoc-2.1 [41,42] to infer functional positioning of the protein.

### 2.4. ADMET Analysis

ADMET properties were predicted using the SWISS-ADME server (http://www.swissadme.ch, accessed on 5 December 2025) [43]. Compounds were screened according to the Tice rule [44], which considers molecular weight, hydrogen-bond donors and acceptors, LogP, and rotatable bonds. This screening step was applied to prioritise compounds with favourable insecticide likeness prior to docking analysis.

### 2.5. Molecular Docking Analysis

Molecular docking was performed using the CB-Dock2 server (https://cadd.labshare.cn/cb-dock2/, accessed on 10 December 2025) [45], which integrates cavity detection with AutoDock Vina. Blind docking was employed because precise active-site boundaries could not be reliably defined in the homology-modelled cytochrome P450 structure, allowing unbiased exploration of potential binding cavities. Docking poses were ranked based on Vina binding affinity scores, and interacting residues were analysed.

### 2.6. Molecular Dynamics Simulation

Molecular dynamics (MD) simulations were conducted using the LiteFold web server (https://www.litefold.in/, accessed on 20 December 2025). Protein–ligand complexes were prepared using the AMBER14 force field with TIP3P explicit solvation and 0.15 M ionic strength. After equilibration, a 20 ns production simulation was performed under NPT conditions. The 20 ns simulation timeframe was selected to balance computational efficiency with sufficient conformational sampling for preliminary stability assessment, as commonly applied in early-stage in silico screening studies. RMSD, RMSF, and radius of gyration (Rg) analyses were used to evaluate structural stability.

### 2.7. Toxicological Safety, Ecological and Environmental Risk Assessment

Toxicity predictions were carried out using ProTox-3.0 (https://tox.charite.de, accessed on 5 January 2026) [46], which estimates LD_50_ values and toxicity classes. Environmental and ecological risk assessments were performed using the ChemFREE platform [47], covering predicted toxicity to aquatic and terrestrial organisms, biodegradability, and environmental persistence. These predictions were treated as indicative screening tools rather than definitive safety assessments, highlighting potential risks that require controlled application and experimental validation. As these data are derived solely from computational prediction models and not from in vitro or in vivo experiments, they were used as preliminary screening indicators. Although predicted trends are generally consistent with previously reported bioassay findings for several compounds, experimental validation is required to confirm toxicity, ecological risk, and environmental fate prior to practical application.

## 3. Results

### 3.1. Retrieval and Characterisation of Ligands

A total of 28 bioactive botanical compounds with reported termiticidal, antifeedant, or repellent activity were retrieved from the PubChem database (Supplementary Table S1). These ligands represent diverse chemical classes, including diterpenoids, sesquiterpenes, flavonoids, phenolics, and monoterpenoids. All compounds were downloaded in SDF format using their respective PubChem CIDs, and key chemical information (IUPAC name, molecular formula, molecular weight, and 3D structure) was obtained from the database.

### 3.2. Protein Sequence Retrieval, Homology Modeling, and Structural Characterization

**Figure 1:**
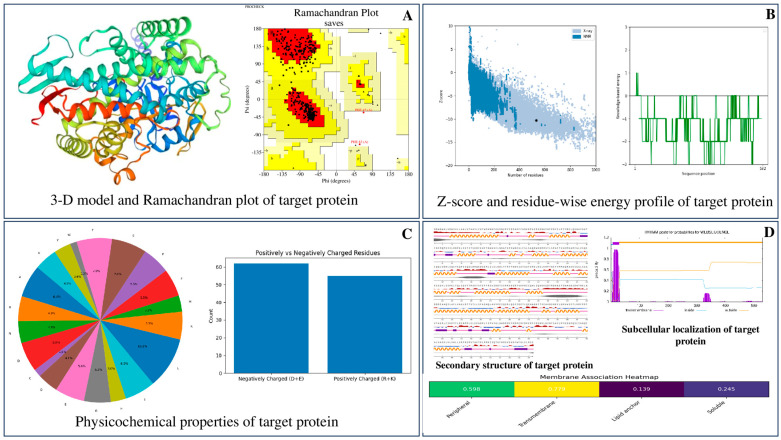
Structural validation and bioinformatic characterization of the target cytochrome P450 protein of *Coptotermes* spp. (**A**) The predicted 3D model and Ramachandran plot confirm good stereochemical quality. (**B**) ProSA Z-score and residue-wise energy profile indicate overall structural reliability. (**C**) Physicochemical properties, including amino acid composition and charge distribution, are presented. (**D**) Secondary structure elements, transmembrane topology, and subcellular localization analysis support the membrane-associated nature of the protein.

As the three-dimensional structure of cytochrome P450 from *C. formosanus* was unavailable in the PDB, its amino acid sequence (Accession No. AFH78148.1) was retrieved from NCBI and used for homology modeling. The predicted model exhibited high stereochemical quality, with 96.42% of residues in favored and allowed regions of the Ramachandran plot and a ProSA Z-score of −10.27, indicating a reliable structure (Figure 1). Physicochemical analysis revealed that the protein comprises 532 amino acids with a molecular weight of 60.47 kDa. Leucine (10.2%), serine (7.0%), threonine (7.0%), alanine (6.4%), and isoleucine (6.0%) were the most abundant residues. The theoretical pI (6.17) indicated an acidic protein, supported by a higher number of negatively charged residues (Asp + Glu = 62) than positively charged ones (Arg + Lys = 55). The protein showed good stability, with an aliphatic index of 82.52 and a GRAVY value of −0.289, reflecting a thermally stable and moderately hydrophilic nature.

Secondary structure and surface accessibility predictions (NetSurfP-3.0) indicated a typical distribution of α-helices, β-strands, and coils, with distinct exposed and buried regions (Figure 1). Subcellular localization analysis (DeepLoc-2.1) predicted endoplasmic reticulum localization, supported by the presence of a signal peptide and a transmembrane domain. Transmembrane topology predictions from TMHMM v2.0 and HMMTOP 2.0 consistently identified an N-terminal transmembrane helix, confirming that cytochrome P450 of *C. formosanus* is a membrane-associated, ER-localized protein with structural features characteristic of P450 enzymes.

### 3.3. Insecticide Likeness Evaluation

The insecticide likeness analysis of the 28 botanical compounds based on the Tice rule revealed that 27 compounds (96.55%) fully complied with all physicochemical thresholds, indicating high suitability as potential insecticidal agents (Table 1; Supplementary Figure S1). Molecular weight values for all molecules ranged between 136.23 g/mol (Limonene) and 378.42 g/mol (Cnicin), remaining well below the recommended cut-off of 500 g/mol. Hydrogen bond donor counts varied from 0 to 3, with the majority of terpenoids such as Vulgarone B, α-gurjunene, Alloaromadendrene, Humulene, Caryophyllene, Limonene, and Piperitone possessing zero donors, reflecting strong hydrophobicity and enhanced membrane permeability. Hydrogen bond acceptor values remained within the acceptable limit (<12), ranging from 0 to 7 across all compounds. Only Quercetin exceeded the donor threshold (5 donors), resulting in a single violation, while all other 28 compounds exhibited no violations. Rotatable bond numbers ranged between 0 and 11, with all molecules remaining within the permissible structural flexibility limit (<12). Compounds such as Gingerol (10), Shogoal (11), Manool (4), and Geraniol (4) exhibited moderate flexibility, which is favourable for optimal binding conformations within the active site of protein targets. Lipophilicity (LogP) values ranged from 0.5 (Citral) to 5.65 (α-gurjunene and Alloaromadendrene), with 27 compounds falling below the recommended threshold (<5). α-gurjunene and Alloaromadendrene slightly exceeded the threshold (*Log p* = 5.65); however, these compounds were retained for further analysis due to their reported termiticidal activity and favourable performance in other key physicochemical and insecticide likeness parameters, resulting in no overall Tice-rule violation.

### 3.4. Molecular Docking Analysis of Botanical Compounds with Cytochrome P450

**Figure 2:**
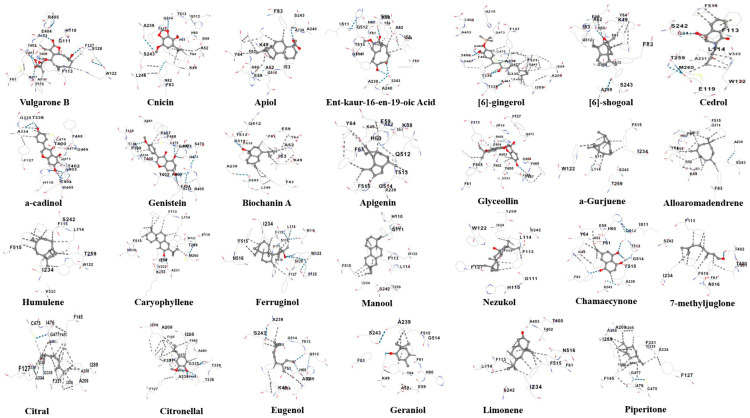
Molecular docking interaction profiles of the botanical bioactive compounds with Coptotermes cytochrome P450. The panels illustrate binding orientations and key hydrogen-bonding and hydrophobic interactions of each ligand within the active site, highlighting a shared binding pocket and residue network involved in ligand stabilization (The interactions between the ligand and the targeted protein are represented by colored dots as follows: the **orange dot** indicates a cation–π interaction; the **green dot** represents π–π stacking; the **grey dot** denotes hydrophobic contact; the **yellow dot** corresponds to ionic interactions; the **blue dot** indicates hydrogen bonds; and the **light blue dot** represents weak hydrogen bonds).

Molecular docking of the 27 botanical compounds with cytochrome P450 of *C. formosanus* showed a wide range of binding affinities, with docking scores from −4.5 to −9.1 kcal/mol, indicating generally favorable ligand–protein interactions (Supplementary Table S2; Figure 2). Glyceollin displayed the strongest binding affinity (−9.1 kcal/mol), followed by Cnicin (−8.7 kcal/mol), ent-kaur-16-en-19-oic acid (−8.2 kcal/mol), Biochanin A (−8.0 kcal/mol), and Ferruginol (−8.0 kcal/mol), suggesting high complex stability and strong inhibitory potential. Several compounds showed moderate binding affinity, including Manool (−7.7 kcal/mol), Genistein (−7.6 kcal/mol), Caryophyllene and Alloaromadendrene (−7.5 kcal/mol), Vulgarone B (−7.4 kcal/mol), and Humulene, Chamaecynone, and Cedrol (−7.2 kcal/mol). Most ligands occupied a common binding pocket comprising residues such as LYS49, ALA52, ILE53, GLU59, HIS60, PHE61, TYR64, HIS110, SER112–LEU114, TRP122, PHE127, VAL232, ILE234, SER242–SER243, THR259, ALA334, ASP337, THR338, THR400–ALA403, GLU404, ARG405, PRO467, PHE468, GLY469, CYS475, ILE476, and GLY514–ASN516, indicating a shared stabilization site. Glyceollin interacted extensively with more than 40 active-site residues, explaining its highest binding energy, while Cnicin formed numerous hydrogen bonds and hydrophobic contacts, supporting its strong affinity. In contrast, compounds with lower affinities, such as Limonene (−4.5 kcal/mol), Geraniol (−5.4 kcal/mol), Citral (−5.5 kcal/mol), and Piperitone (−5.6 kcal/mol), showed limited interactions, mainly hydrophobic, consistent with their simpler structures. Overall, structurally complex flavonoids and terpenoids exhibited superior binding compared to monoterpenes, highlighting Glyceollin, Cnicin, Biochanin A, Ferruginol, ent-kaur-16-en-19-oic acid, and Manool as promising cytochrome P450 inhibitors. Docking analyses of chlorpyrifos and imidacloprid were included as reference standards for comparison (Supplementary Figure S2).

### 3.5. Toxicity Prediction of Bioactive Botanical Compounds

**Figure 3:**
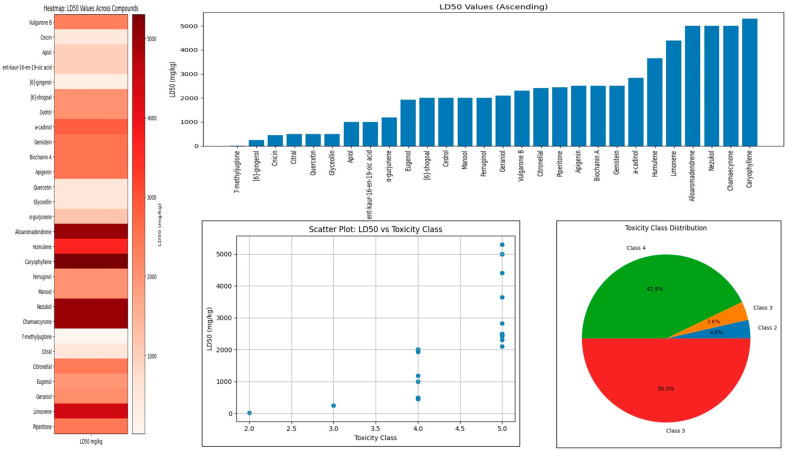
Toxicological profiling of botanical compounds based on predicted mammalian LD_50_ values. The figure integrates heatmap, ranked LD_50_ distribution, LD_50_–toxicity class relationship, and class frequency analysis, illustrating that most compounds fall within lower-toxicity classes with comparatively high LD_50_ values.

The predicted oral acute toxicity (LD_50_) classified the 27 botanical compounds into Toxicity Classes II–V, indicating a wide but predominantly moderate toxicity range (Table 2; Figure 3). LD_50_ values varied from 16 to 5300 mg/kg, reflecting substantial chemical diversity. Most compounds (22/29; 78.57%) belonged to Toxicity Class V (LD_50_ = 2000–5000 mg/kg; “may be harmful if swallowed”), including Vulgarone B, Genistein, Biochanin A, Apigenin, Humulene, Limonene, and Caryophyllene, suggesting relatively low acute toxicity and better environmental safety. Six compounds (6/29; 21.42%) were categorized as Toxicity Class IV (LD_50_ = 300–2000 mg/kg; “harmful if swallowed”), such as Cnicin, Glyceollin, Citral, Apiol, ent-kaur-16-en-19-oic acid, and [6]-shogoal, indicating moderate toxicity. Only one compound, [6]-gingerol, fell into Toxicity Class III (LD_50_ = 250 mg/kg; “toxic if swallowed”). In contrast, 7-methyljuglone showed the highest toxicity (LD_50_ = 16 mg/kg; Class II, “fatal if swallowed”), raising concerns regarding mammalian safety. The least toxic compounds—Caryophyllene, Alloaromadendrene, and Nezukol—approached or exceeded the upper limit of Class V (5000–5300 mg/kg). Overall, most botanicals exhibited low to moderate acute toxicity, supporting their potential as environmentally compatible bioinsecticides, with only a few requiring careful risk consideration.

### 3.6. Ecological and Environmental Toxicity Assessment

**Figure 4:**
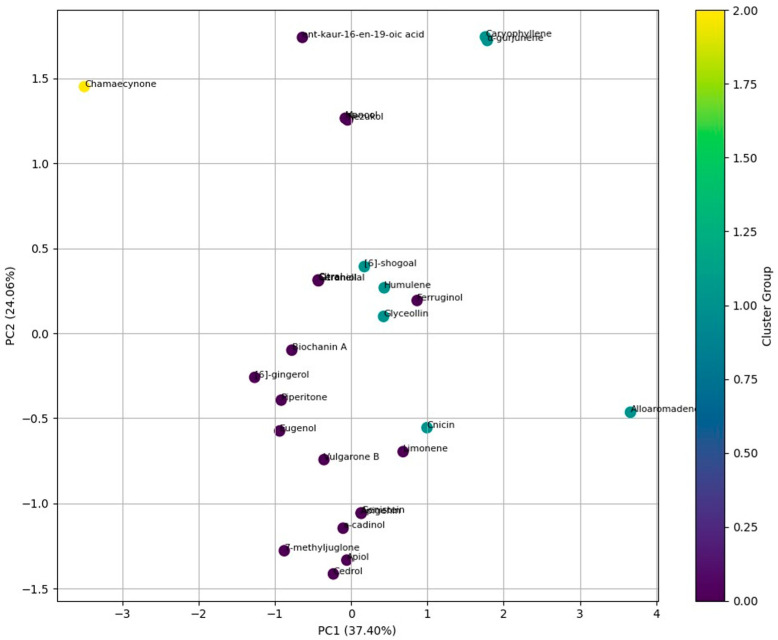
PCA and cluster analysis of environmental persistence (air, water, soil, and sediment) of botanical compounds. PC1 and PC2 explain the major variance in persistence profiles, grouping compounds with similar degradation and stability patterns and highlighting distinct clusters of low- and high-persistence botanicals.

The ecological toxicity assessment across nine non-target organisms showed a clear pattern of selective safety and risk among the 27 botanical compounds (Table 3). Most compounds were non-toxic to terrestrial vertebrates, exhibiting LD_50_ values > 500 mg/kg for mammals and >2000 mg/kg for birds, indicating safety for mallard duck, bobwhite quail, and zebra finch. Compounds such as α-gurjunene, Alloaromadendrene, Humulene, Caryophyllene, Nezukol, Limonene, and Piperitone were consistently non-toxic to all tested terrestrial organisms. In contrast, Cnicin, [6]-gingerol, Glyceollin, and 7-methyljuglone showed mammalian or avian toxicity. Most botanicals were also safe to pollinators, although Cnicin, [6]-gingerol, Glyceollin, and Chamaecynone showed predicted bee toxicity. Conversely, aquatic organisms were highly sensitive, with nearly all compounds showing toxicity to algae, fish, daphnia, and mysids, except for limited cases such as Vulgarone B and Glyceollin (daphnia) and Apiol (mysids). Overall, the compounds appear environmentally compatible for terrestrial use but pose significant risks to aquatic ecosystems, necessitating careful application. Environmental fate analysis revealed substantial variability in persistence across compartments (Figure 4). Most compounds showed low atmospheric persistence (0.008–11.12 h), with exceptions such as Cnicin (17.68 h) and Chamaecynone (391.34 h). In water, persistence ranged from 3.50 to 25.66 days, with α-gurjunene and Alloaromadendrene being the most stable. Soil persistence varied widely (7.0–101.04 days), with rapid degradation of [6]-gingerol, Citral, Geraniol, and Piperitone, but prolonged persistence of Cnicin, [6]-shogoal, Glyceollin, and α-gurjunene. Sediment persistence ranged from 13 to 532 days, with Alloaromadendrene, Caryophyllene, and Glyceollin showing the highest stability. Biodegradability analysis identified 13 compounds as biodegradable, including [6]-gingerol, Genistein, Biochanin A, Apigenin, Citral, Eugenol, Geraniol, Limonene, and Piperitone, whereas 16 compounds were non-biodegradable, consistent with their higher soil and sediment persistence.

### 3.7. Ligand-Induced Stabilization of Cytochrome P450

**Figure 5:**
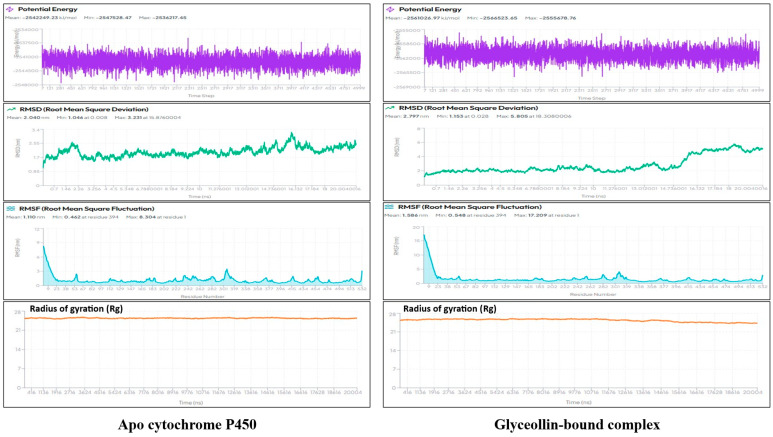
Molecular dynamics simulation profiles of apo cytochrome P450 and glyceollin-bound complex. Comparative analyses of potential energy, RMSD, RMSF, and radius of gyration demonstrate enhanced energetic stability, controlled flexibility, and slight structural compaction of cytochrome P450 upon Glyceollin binding.

Given the strong binding affinity of Glyceollin toward cytochrome P450 in molecular docking, a 20 ns molecular dynamics simulation was performed to evaluate the dynamic stability and conformational behaviour of the protein–ligand complex relative to the apo protein (Figure 5). A marked stabilizing effect was observed upon ligand association. The apo cytochrome P450 exhibited a mean potential energy of −2,542,249.23 kJ/mol, with fluctuations spanning −2,547,528.47 to −2,536,217.45 kJ/mol, reflecting a moderately stable but thermodynamically flexible system. In contrast, the Glyceollin-bound complex demonstrated a substantially lower mean potential energy of −2,561,026.97 kJ/mol, with a narrower distribution (−2,566,523.65 to −2,555,678.76 kJ/mol). This ~18,778 kJ/mol decrease indicates pronounced thermodynamic stabilization driven by ligand–protein interactions. The tighter fluctuation range further suggests reduced internal strain and improved energetic uniformity in the complex. The structural stability of the systems exhibited clear differences. The unbound protein maintained a mean RMSD of 2.040 nm, stabilizing early around 1.7–2.2 nm, indicative of a relatively stable backbone with limited large-scale motions. Conversely, the protein-ligand complex showed a higher mean RMSD of 2.797 nm with values ranging from 1.153 to 5.805 nm, reflecting increased global flexibility following ligand engagement. Although stable during the initial 8–10 ns, the complex displayed gradual RMSD elevation in the later stages, suggesting ligand-induced structural adjustments consistent with an induced-fit mechanism. Importantly, the RMSD profile lacked abrupt spikes, confirming that the complex remained dynamically stable throughout the simulation.

Residue-level dynamics further highlighted ligand-induced modulation. The apo protein exhibited an average RMSF of 1.110 nm, with maximum fluctuations at the N-terminal residues (up to 8.304 nm) followed by consistently low fluctuations (<1.5 nm) across most of the backbone. Ligand binding resulted in higher global flexibility, with the complex showing a mean RMSF of 1.586 nm and an expanded fluctuation range (up to 17.209 nm at residue 1). Increased mobility was primarily localized to the terminal and loop regions (residues 250–310 and 420–470), while the active-site and catalytic core remained comparatively stable (<1 nm), indicating that Glyceollin binding promotes local conformational rearrangements without compromising the functional structural integrity. The radius of gyration (Rg) trajectories revealed that both systems remained compact and structurally intact throughout the simulation. The apo protein maintained a mean Rg of 25.405 Å (range: 25.039–25.856 Å), whereas the protein–ligand complex exhibited a slightly lower mean Rg of 25.369 Å (range: 24.158–26.114 Å). A modest decrease in Rg during the latter half of the simulation for the complex indicates subtle structural compaction upon ligand binding, likely facilitated by enhanced intramolecular contacts. This increased compactness is consistent with the observed lower potential energy and supports the conclusion that ligand binding confers additional structural stability to the protein. Collectively, the MD simulation illustrates that Glyceollin binding enhances the thermodynamic and conformational stability of cytochrome P450. While inducing modest increases in backbone and loop flexibility, the ligand stabilizes the global fold, improves energetic uniformity, and promotes slight structural compaction-features consistent with a stable and favourable protein–ligand interaction.

## 4. Discussion

Botanical insecticides have long been recognized as environmentally compatible alternatives to synthetic pesticides, whose indiscriminate and prolonged use has resulted in phytotoxicity, mammalian toxicity, pesticide resistance, and ecological disruption [48]. Plants naturally synthesize a diverse range of secondary metabolites, including terpenoids, phenolics, flavonoids, and alkaloids, which function as defensive compounds against herbivores and other biotic stresses [49,50]. Despite extensive traditional use, systematic scientific investigation of botanicals for termite management has remained limited, with most prior efforts focused on empirical bioassays rather than mechanistic understanding or molecular target identification [48]. Consequently, the molecular basis underlying the termiticidal activity of many plant-derived compounds has remained insufficiently explored [51,52,53]. A critical prerequisite for mechanistic inference in silico is the reliability of the target protein structure. In the present study, cytochrome P450 from *C. formosanus* was modeled and validated using multiple stereochemical and energetic criteria. The model showed 96.42% of residues within favored and allowed regions of the Ramachandran plot and a ProSA Z-score of −10.27, comparable to experimentally resolved proteins of similar size. These metrics indicate a structurally sound model suitable for docking and simulation studies. Nevertheless, uncertainties inherent to homology modeling, particularly in flexible loop regions and precise active-site geometry, were considered during interpretation and justified the use of blind docking rather than site-restricted approaches.

The integrated computational framework applied here evaluated 28 bioactive botanical compounds with particular emphasis on cytochrome P450 inhibition in *C. formosanus*. Cytochrome P450 enzymes play a central role in termite survival by mediating detoxification of plant allelochemicals, xenobiotics, and metabolic by-products generated during lignocellulose digestion. Furthermore, enhanced P450 activity has been associated with metabolic tolerance and resistance in insects. Inhibition of this enzyme system is therefore expected to compromise detoxification pathways and disrupt metabolic homeostasis. Compounds previously reported as termiticidal in laboratory bioassays, including Cnicin, Glyceollin, and Apiol [22], exhibited strong docking affinities in the present study (−8.7, −9.1, and ~−7.0 kcal/mol, respectively), providing quantitative mechanistic support for their observed biological activity. Similarly, phenolic and flavonoid compounds such as genistein, biochanin A, apigenin, quercetin, and glyceollin, which have been reported to induce mortality or reduce fecundity in *C. formosanus* [26], displayed docking scores consistently stronger than −7.5 kcal/mol. Notably, Glyceollin emerged as the strongest binder (−9.1 kcal/mol) and formed extensive interactions with conserved catalytic residues, suggesting a high likelihood of effective enzyme inhibition. In contrast, monoterpenes such as limonene, geraniol, citral, and piperitone, primarily reported as repellents rather than lethal agents [32,33,52,53]—showed weaker binding energies (−4.5 to −5.6 kcal/mol), reflecting their lower molecular complexity and limited functional group diversity. This quantitative trend supports the biological distinction between repellent and inhibitory activity.

Insecticide likeness and ADMET screening further refined compound prioritization. Twenty-seven of the 28 compounds satisfied all Tice criteria, indicating favorable molecular weight (<500 g/mol), hydrogen-bonding capacity, flexibility, and lipophilicity. Although α-gurjunene and alloaromadendrene slightly exceeded the LogP threshold (5.65), they were retained due to their documented termiticidal activity and strong docking performance (−7.5 kcal/mol), highlighting the importance of integrating empirical evidence with rule-based screening. Terpenoids constituted another major class of interest. Sesquiterpenes such as alloaromadendrene, humulene, and caryophyllene, previously isolated from Dipterocarpus resins and reported as toxic to Southeast Asian termites [27,28,50], exhibited moderate-to-strong binding affinities (−7.2 to −7.5 kcal/mol). Diterpenes such as ferruginol, manool, and nezukol, earlier isolated from Taxodium distichum and reported as antifeedants against *C. formosanus* [29,51], emerged as among the strongest binders, with ferruginol showing a docking score of −8.0 kcal/mol. These quantitative results reinforce earlier bioassay findings and suggest detoxification interference as a plausible mode of action. Essential oil constituents, including citral, citronellal, geraniol, and limonene, displayed acceptable insecticide-likeness and low predicted mammalian toxicity, consistent with their widespread use and reported safety [32,33,52,53].

Molecular dynamics simulations provided dynamic validation of docking predictions. The Glyceollin–P450 complex exhibited a marked reduction in mean potential energy (~18,778 kJ/mol lower than the apo protein), indicating pronounced thermodynamic stabilization. While the protein–ligand complex showed increased RMSD and RMSF values relative to the apo form, these changes were localized primarily to terminal and loop regions, with the catalytic core remaining stable. The radius of gyration decreased slightly upon ligand binding (25.405 Å to 25.369 Å), indicating subtle structural compaction. Together, these data support a stable induced-fit interaction rather than transient binding. Toxicological predictions further aligned with experimental trends. Most compounds (≈75%) were classified under Toxicity Class V (LD_50_ > 2000 mg/kg), including limonene (4400 mg/kg) and caryophyllene (5300 mg/kg), which are widely regarded as safe botanicals. In contrast, highly bioactive compounds such as 7-methyljuglone exhibited very low predicted LD_50_ values (16 mg/kg), consistent with their known biological potency. Ecological assessments revealed generally low risk to mammals, birds, and pollinators but consistently high predicted toxicity toward aquatic organisms, corroborating earlier warnings regarding botanical pesticide use near aquatic systems [51,54].

Environmental fate analysis highlighted an important efficacy–persistence trade-off. Rapidly degradable compounds such as citral, citronellal, geraniol, and limonene exhibited short half-lives in soil and water, whereas hydrophobic compounds like alloaromadendrene, α-gurjunene, and ferruginol showed prolonged persistence in soil (up to ~101 days) and sediment (up to ~532 days) [55,56]. These quantitative differences underscore the need for compound-specific risk assessment rather than generalized assumptions of environmental safety. Overall, this study integrates docking, molecular dynamics, toxicity prediction, and environmental fate analysis to provide mechanistic and quantitative insight into botanical inhibition of cytochrome P450 in *C. formosanus*. While the findings are predictive in nature, the strong numerical agreement between computational outputs and experimentally reported biological behavior supports the reliability of the approach. Nevertheless, these results should be regarded as hypothesis generating, and experimental validation through enzyme inhibition assays, termite bioefficacy trials, and comprehensive ecotoxicological studies remains essential for translating these candidates into sustainable termite management solutions.

## 5. Conclusions

This study applied an integrated in silico framework to evaluate twenty-eight plant-derived bioactive compounds for their potential to inhibit cytochrome P450 in *Coptotermes* spp., a key enzyme involved in termite detoxification and metabolic resilience. Screening based on insecticide likeness criteria showed that 27 of the 28 compounds satisfied all Tice parameters, indicating favourable physicochemical suitability for bioinsecticidal development. Molecular docking analysis identified Glyceollin (−9.1 kcal/mol) as the strongest binder, followed by Cnicin, Biochanin A, Ferruginol, and ent-kaur-16-en-19-oic acid, all of which formed stable interactions with conserved residues of the cytochrome P450 active site. Molecular dynamics simulations further supported the stabilizing effect of Glyceollin binding, as evidenced by lower potential energy, slight structural compaction, and stable RMSD, RMSF, and radius of gyration profiles compared with the apo protein. Toxicological predictions suggested that more than 75% of the compounds fall within low-toxicity categories (Toxicity Class V), implying limited acute risk. Ecological assessments indicated relatively low predicted toxicity to terrestrial non-target organisms, including mammals, birds, and pollinators. However, several compounds exhibited notable predicted toxicity toward aquatic organisms, highlighting an important ecological limitation and the need for controlled application. Environmental fate analysis revealed substantial variability in biodegradability and persistence, with compounds such as [6]-gingerol, genistein, citral, citronellal, geraniol, and limonene showing comparatively rapid degradation. Overall, the study identifies Glyceollin, along with Cnicin, Biochanin A, Ferruginol, and ent-kaur-16-en-19-oic acid, as promising preliminary candidates for cytochrome P450 targeting in termites. Nevertheless, these findings are predictive in nature, and comprehensive experimental validation through biochemical, toxicological, and field-based studies is essential before practical application in sustainable termite management.

## Figures and Tables

**Table 1:** Physicochemical properties and insecticide likeness evaluation of bioactive botanical compounds based on the Tice rule.

Pubchem Id	Bioactive Compounds	Molecular Weight (<500g/mol)	H-Bond Donors (<3)	H-Bond Acceptor (<12)	No. of Rotatable Bonds (<12)	*Log p* (<5)	No. of Violations (Tice Rule)
530428	Vulgarone B	218.33	0	1	0	3.56	0
5281435	Cnicin	378.42	3	7	6	0.92	0
10659	Apiol	222.24	0	4	4	1.4	0
73062	ent-kaur-16-en-19-oic acid	302.45	1	2	1	4.63	0
442793	[6]-gingerol	294.39	2	4	10	2.14	0
5281794	[6]-shogoal	348.55	0	3	11	3.6	0
65575	Cedrol	222.37	1	1	0	3.81	0
10398656	a-cadinol	222.37	1	1	1	3.67	0
5280961	Genistein	270.24	3	5	1	0.52	0
5280373	Biochanin A	284.26	2	5	2	0.77	0
5280443	Apigenin	270.24	3	5	1	0.52	0
5280343	Quercetin	302.24	5	7	1	1.54	1
162807	Glyceollin	338.35	2	5	0	1.9	0
15560275	α-gurjunene	204.35	0	0	0	5.65	0
10899740	Alloaromadendrene	204.35	0	0	0	5.65	0
5281520	Humulene	204.35	0	0	0	4.53	0
5281515	Caryophyllene	204.35	0	0	0	4.53	0
442027	Ferruginol	286.45	1	1	1	4.92	0
3034394	Manool	290.48	1	1	4	4.75	0
13969544	Nezukol	290.48	1	1	1	4.86	0
193451	Chamaecynone	290.48	1	1	10	4.92	0
26905	7-methyljuglone	202.29	0	1	0	3.29	0
638011	Citral	188.18	1	3	0	0.5	0
7794	Citronellal	152.23	0	1	4	2.49	0
3314	Eugenol	152.25	0	1	5	2.52	0
637566	Geraniol	154.25	1	1	4	2.59	0
22311	Limonene	136.23	0	0	1	3.27	0
6987	Piperitone	152.23	0	1	1	2.2	0

**Table 2:** Predicted acute oral toxicity (LD_50_) and corresponding toxicity classes of bioactive botanical compounds.

Bioactive Compounds	Predicted LD_50_	Predicted Toxicity Class
Vulgarone B	2300 mg/kg	5
Cnicin	452 mg/kg	4
Apiol	1000 mg/kg	4
ent-kaur-16-en-19-oic acid	1000 mg/kg	4
[6]-gingerol	250 mg/kg	3
[6]-shogoal	2000 mg/kg	4
Cedrol	2000 mg/kg	4
a-cadinol	2830 mg/kg	5
Genistein	2500 mg/kg	5
Biochanin A	2500 mg/kg	5
Apigenin	2500 mg/kg	5
Glyceollin	500 mg/kg	4
α-gurjunene	1190 mg/kg	4
Alloaromadendrene	5000 mg/kg	5
Humulene	3650 mg/kg	5
Caryophyllene	5300 mg/kg	5
Ferruginol	2000 mg/kg	4
Manool	2000 mg/kg	4
Nezukol	5000 mg/kg	5
Chamaecynone	5000 mg/kg	5
7-methyljuglone	16 mg/kg	2
Citral	500 mg/kg	4
Citronellal	2420 mg/kg	5
Eugenol	1930 mg/kg	4
Geraniol	2100 mg/kg	5
Limonene	4400 mg/kg	5
Piperitone	2450 mg/kg	5

Class I: fatal if swallowed (LD50 ≤ 5); Class II: fatal if swallowed (5 < LD50 ≤ 50); Class III: toxic if swallowed (50 < LD50 ≤ 300); Class IV: harmful if swallowed (300 < LD50 ≤ 2000); Class V: may be harmful if swallowed (2000 < LD50 ≤ 5000); Class VI: non-toxic (LD50 > 5000).

**Table 3:** Ecological toxicity classification of the evaluated bioactive compounds across nine non-target organisms.

Bioactive Compounds	Mammal Toxicity	Bee Toxicity	Mallard Duck Toxicity	Bobwhite Quail Toxicity	Zebra Finch Toxicity	Algae Toxicity	Fish Toxicity	Daphnia Toxicity	Mysid Toxicity
Vulgarone B									
Cnicin									
Apiol									
ent-kaur-16-en-19-oic acid									
[6]-gingerol									
[6]-shogoal									
Cedrol									
a-cadinol									
Genistein									
Biochanin A									
Apigenin									
Glyceollin									
α-gurjunene									
Alloaromadendrene									
Humulene									
Caryophyllene									
Ferruginol									
Manool									
Nezukol									
Chamaecynone									
7-methyljuglone									
Citral									
Citronellal									
Eugenol									
Geraniol									
Limonene									
Piperitone									
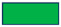 Non-toxic; 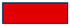 Toxic									

Mammal toxicity: LD_50_ > 500 mg/kg—Non-toxic, LD_50_ < 500 mg/kg –toxic; Mallard duck toxicity: LD_50_ > 2000 mg/kg—Non-toxic, LD_50_ < 2000 mg/kg–toxic; Bobwhite quail toxicity: LD_50_ > 2000 mg/kg—Non-toxic, LD_50_ < 2000 mg/kg–toxic; Zebra finch toxicity: LD_50_ > 2000 mg/kg—Non-toxic, LD_50_ < 2000 mg/kg–toxic; Bee toxicity: LD_50_ > 11 µg/bee—Non-toxic, LD_50_ < 11 µg/bee–toxic; Algae toxicity: IC_50_ > 1 mg/L–Non-toxic, IC_50_ < 1 mg/L–toxic; Fish toxicity: LC_50_ > 100 mg/L–Non-toxic, LC_50_ < 100 mg/L –toxic; Daphnia toxicity: LC_50_ > 100 mg/L—Non-toxic, LC_50_ < 100 mg/L–toxic; Mysid toxicity: LC_50_ > 10 mg/L–Non-toxic, LC_50_ < 10 mg/L–toxic.

## Data Availability

All the data used in this study were sourced from public databases.

## Supplementary Materials

The following supporting information can be downloaded at: https://www.mdpi.com/article/10.3390/plants15040581/s1, Figure S1. Comparative radar plot of physicochemical properties of the botanical bioactive compounds used for ligand screening. Figure S2. Molecular docking interactions of cytochrome P450 with chlorpyrifos (left) and imidacloprid (right), illustrating ligand orientation within the active site. Both insecticides showed comparable binding affinity (−6.2 kcal/mol) and interacted with key catalytic and surrounding residues of cytochrome P450. Table S1. List of botanical bioactive compounds retrieved from PubChem. Table S2. Molecular docking scores and interaction profiles of bioactive botanical compounds with cytochrome P450 of *Coptotermes formosanus*.
