# Supplementary material for: In Silico Screening of Plant-Derived Termiticidal Compounds Targeting Cytochrome P450 in Coptotermes spp. (Blattodea: Rhinotermitidae) for Sustainable Termite Management
Source: Plants (Basel). 2026 Feb 12;15(4):581. doi: 10.3390/plants15040581 (PMC12944165; doi:10.3390/plants15040581)
Supplement: Supplementary file 1 [file plants-15-00581-s001.zip › plants-4135869-supplementary.pdf]

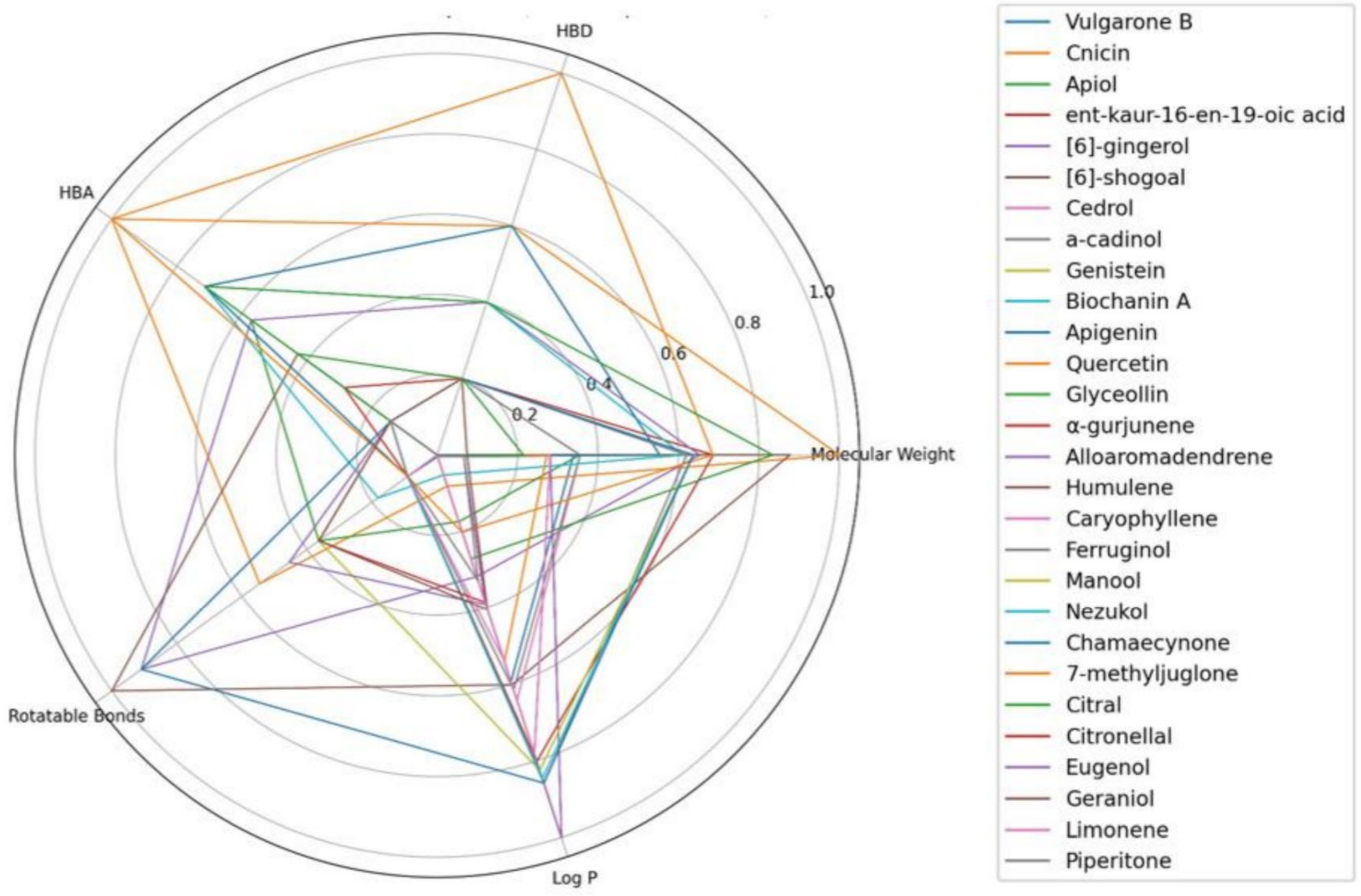

Supplementary Figure S1. Comparative radar plot of physicochemical properties of the botanical bioactive compounds used for ligand screening

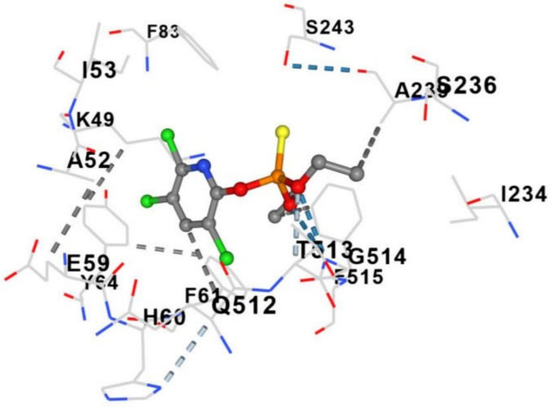

Cytochrome p450-Chlorpyrifos

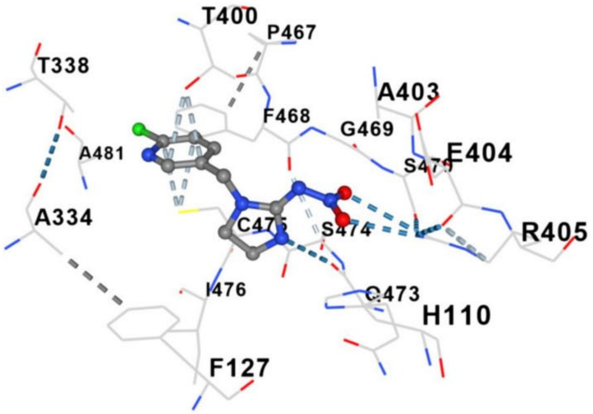

Cytochrome p450-Imidacloprid

Commercial insecticides	Docking score with cytochrome p450 (kcal/mol)	Contact residues with cytochrome p450
Chlorpyrifos	-6.2	LYS49 ALA52 ILE53 LYS58 GLU59 HIS60 PHE61 TYR64 ASN82 PHE83 HIS110 GLY111 SER112 PHE113 LEU114 TRP122 PHE127 SER128 ILE234 GLY235 SER236 ALA239 ALA240 SER243 LEU246 ALA334 GLY335 THR338 THR339 LEU394 PRO399 THR400 THR402 ALA403 GLU404 ARG405 VAL431 LEU466 PRO467 PHE468 GLY469 SER474 CYS475 ILE476 GLN512 THR513 GLY514 PHE515
Imidacloprid	-6.2	PHE61 HIS110 GLY111 SER112 PHE113 LEU114 ASN116 TRP122 PHE127 SER128 ASN194 THR198 GLN229 VAL232 LYS233 ILE234 SER242 THR259 MET260 ALA334 ASP337 THR338 SER340 THR341 THR400 VAL401 THR402 ALA403 GLU404 ARG405 PHE428 PRO467 PHE468 GLY469 SER470 GLN473 SER474 CYS475 ILE476 ALA481 ILE511 THR513 GLY514 PHE515 ASN516 MET517 SER518 VAL519 PHE523

Supplementary Figure S2. Molecular docking interactions of cytochrome P450 with chlorpyrifos (left) and imidacloprid (right), illustrating ligand orientation within the active site. Both insecticides showed comparable binding affinity (−6.2 kcal/mol) and interacted with key catalytic and surrounding residues of cytochrome P450.

Supplementary Table S1. List of botanical bioactive compounds retrieved from PubChem.

PubChem IDs	Bioactive compounds	Plant Source	IUPAC Names	Chemical Formula	Molecular Weight (g/mol)	Chemical structure-3D	References
530428	Vulgarone B	*Artemisia douglasiana*	2,6,6,11-tetramethyltricyclo[5.4.0.$0^{2,8}$]undec-10-en-9-one	$C_{15}H_{22}O$	218.33 g/mol	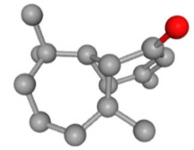	[22]
5281435	Cnicin	*Centaurea maculosa*	[(3aR,4S,6E,10Z,11aR)-10-(hydroxymethyl)-6-methyl-3-methylidene-2-oxo-3a,4,5,8,9,11a-hexahydrocyclodeca[b]furan-4-yl] (3R)-3,4-dihydroxy-2-methylidenebutanoate	$C_{20}H_{26}O_7$	378.4 g/mol	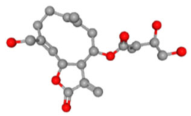	[22]
10659	Apiol	*Ligusticum hultenii*	4,7-dimethoxy-5-prop-2-enyl-1,3-benzodioxole	$C_{12}H_{14}O_4$	222.24 g/mol	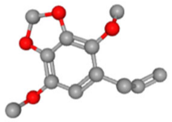	[22]
73062	ent-kaur-16-en-19-oic acid	*Xylopia aethiopica*	(1S,4S,5R,9S,10R,13R)-5,9-dimethyl-14-methylidenetetracyclo[11.2.1.01,10.04,9] hexadecane-5-carboxylic acid	$C_{20}H_{30}O_2$	302.5 g/mol	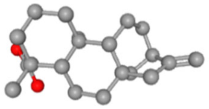	[23]
442793	[6]-gingerol	*Aframomum melegueta*	(5S)-5-hydroxy-1-(4-hydroxy-3-methoxyphenyl)decan-3-one	$C_{17}H_{26}O_4$	294.4 g/mol	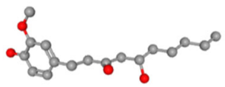	[24]
5281794	[6]-shogoal	*Aframomum melegueta*	(E)-1-(4-hydroxy-3-methoxyphenyl)dec-4-en-3-one	$C_{17}H_{24}O_3$	276.4 g/mol	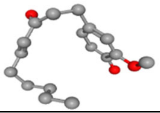	[24]

65575	Cedrol	*Taiwania cryptomerioides*	(1S,2R,5S,7R,8R)-2,6,6,8-tetramethyltricyclo[$5.3.1.0^{1,5}$]undecan-8-ol	$C_{15}H_{26}O$	222.37 g/mol	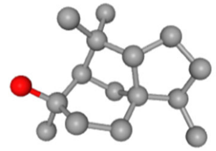	[25]
10398656	a-cadinol	*Taiwania cryptomerioides*	(1R,4S,4aR,8aR)-1,6-dimethyl-4-propan-2-yl-3,4,4a,7,8,8a-hexahydro-2H-naphthalen-1-ol	$C_{15}H_{26}O$	222.37 g/mol	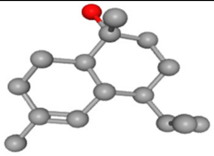	[25]
5280961	Genistein	*Glycine max*	5,7-dihydroxy-3-(4-hydroxyphenyl)chromen-4-one	$C_{15}H_{10}O_5$	270.24 g/mol	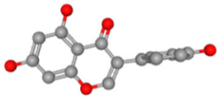	[26]
5280373	Biochanin A	*Trifolium pratense*	5,7-dihydroxy-3-(4-methoxyphenyl)chromen-4-one	$C_{16}H_{12}O_5$	284.26 g/mol	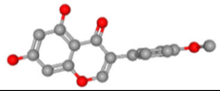	[26]
5280443	Apigenin	*Petroselinum crispum*	5,7-dihydroxy-2-(4-hydroxyphenyl)chromen-4-one	$C_{15}H_{10}O_5$	270.24 g/mol	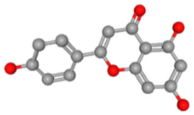	[26]
5280343	Quercetin	*Allium cepa*	2-(3,4-dihydroxyphenyl)-3,5,7-trihydroxychromen-4-one	$C_{15}H_{10}O_7$	302.23 g/mol	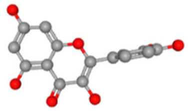	[26]
162807	Glyceollin	*Glycine max*	(2S,10S)-17,17-dimethyl-3,12,18-trioxapentacyclo [11.8.0.02,10.04,9.014,19]henicosa-1(13),4(9),5,7,14(19),15,20-heptaene-6,10-diol	$C_{20}H_{18}O_5$	338.4 g/mol	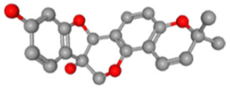	[26]

15560275	α-gurjunene	*Dipterocarpus kerrii*	(1aS,4S,4aS,7bR)-1,1,4,7-tetramethyl-1a,2,3,4,4a,5,6,7b-octahydrocyclopropa[e]azulene	$C_{15}H_{24}$	204.35 g/mol	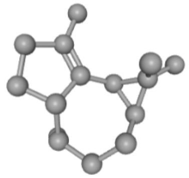	[27]
10899740	Alloaromadendrene	*Dipterocarpus* species	(1aR,4aS,7R,7aR,7bS)-1,1,7-trimethyl-4-methylidene-2,3,4a,5,6,7,7a,7b-octahydro-1aH-cyclopropa[e]azulene	$C_{15}H_{24}$	204.35 g/mol	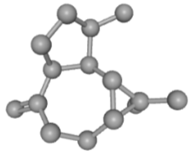	[28]
5281520	Humulene	*Dipterocarpus* species	(1E,4E,8E)-2,6,6,9-tetramethylcycloundeca-1,4,8-triene	$C_{15}H_{24}$	204.35 g/mol	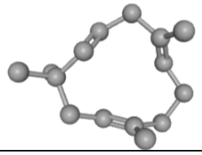	[28]
5281515	Caryophyllene	*Dipterocarpus* species	(1*R*,4*E*,9*S*)-4,11,11-trimethyl-8-methylidenebicyclo[7.2.0]undec-4-ene	$C_{15}H_{24}$	204.35 g/mol	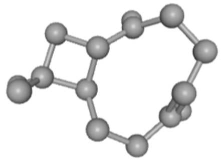	[28]
442027	Ferruginol	*Taxodium distichum*	(4bS,8aS)-4b,8,8-trimethyl-2-propan-2-yl-5,6,7,8a,9,10-hexahydrophenanthren-3-ol	$C_{20}H_{30}O$	286.5 g/mol	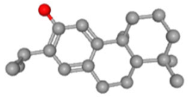	[29]
3034394	Manool	*Taxodium distichum*	(3R)-5-[(1S,4aS,8aS)-5,5,8a-trimethyl-2-methylidene-3,4,4a,6,7,8-hexahydro-1H-naphthalen-1-yl]-3-methylpent-1-en-3-ol	$C_{20}H_{34}O$	290.5 g/mol	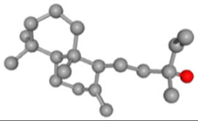	[29]
13969544	Nezukol	*Taxodium distichum*	(4aS,4bR,7S,8aR,10aS)-7-ethenyl-1,1,4a,7-tetramethyl-2,3,4,4b,5,6,8,9,10,10a-decahydrophenanthren-8a-ol	$C_{20}H_{34}O$	290.5 g/mol	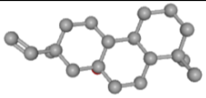	[29]

193451	Chamaecynone	*Chamaecyparis pisifera*	(1S,4aS,7R,8aR)-7-ethynyl-1,4a-dimethyl-1,5,6,7,8,8a-hexahydronaphthalen-2-one	$C_{14}H_{18}O$	202.29 g/mol	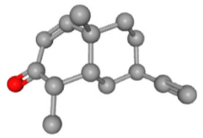	[30,31]
26905	7-methyljuglone	*Diospyros virginiana*	5-hydroxy-7-methylnaphthalene-1,4-dione	$C_{11}H_8O_3$	188.18 g/mol	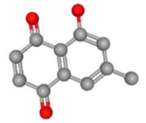	[30,31]
638011	Citral	*Cymbopogon citratus*	(2E)-3,7-dimethylocta-2,6-dienal	$C_{10}H_{16}O$	152.23 g/mol	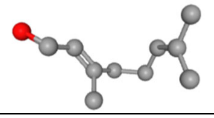	[32,33]
7794	Citronellal	*Cymbopogon nardus*	3,7-dimethyloct-6-enal	$C_{10}H_{18}O$	154.25 g/mol	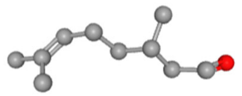	[32,33]
3314	Eugenol	*Syzygium aromaticum*	2-methoxy-4-prop-2-enylphenol	$C_{10}H_{12}O_2$	164.2 g/mol	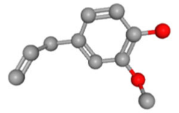	[32,33]
637566	Geraniol	*Cymbopogon martinii*	(2E)-3,7-dimethylocta-2,6-dien-1-ol	$C_{10}H_{18}O$	154.25 g/mol	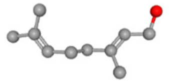	[32,33]
22311	Limonene	*Citrus sinensis*	1-methyl-4-prop-1-en-2-ylcyclohexene	$C_{10}H_{16}$	136.23 g/mol	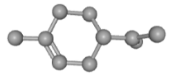	[32,33]
6987	Piperitone	*Mentha longifolia*	3-methyl-6-propan-2-ylcyclohex-2-en-1-one	$C_{10}H_{16}O$	152.23 g/mol	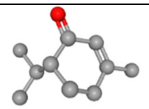	[32,33]

Supplementary Table S2. Molecular docking scores and interaction profiles of bioactive botanical compounds with cytochrome P450 of *Coptotermes formosanus*.

**Bioactive compounds**	**Docking score with cytochrome p450 (kcal/mol)**	**Contact residues with cytochrome p450**
Vulgarone B	-7.4	LYS49 ALA52 ILE53 LYS58 GLU59 HIS60 PHE61 TYR64 PHE83 PHE113 ILE234 ALA239 SER242 SER243 PRO399 THR400 VAL401 THR402 GLU404 ILE511 GLN512 THR513 GLY514 PHE515 ASN516
Cnicin	-8.7	LYS49 ALA52 ILE53 GLU59 HIS60 PHE61 TYR64 ASN82 PHE83 HIS110 GLY111 SER112 PHE113 LEU114 ASN116 TRP122 SER125 PHE127 SER128 ALA231 VAL232 ILE234 SER236 ALA239 ALA240 SER242 SER243 LEU246 THR259 MET260 SER261 VAL333 ALA334 ASP337 THR338 PRO399 THR400 VAL401 THR402 ALA403 GLU404 ARG405 PHE428 VAL431 LEU466 PRO467 PHE468 GLY469 SER470 GLN473 SER474 CYS475 ILE511 GLN512 THR513 GLY514 PHE515 ASN516 MET517
Apiol	-5.7	LYS49 ALA52 ILE53 LYS58 GLU59 HIS60 PHE61 TYR64 ASN82 PHE83 HIS110 TRP122 PHE127 VAL232 ALA239 SER243 LEU246 PHE331 LEU332 VAL333 ALA334 GLY335 LEU336 ASP337 THR338 THR339 PRO399 THR400 THR402 ALA403 ARG405 VAL431 LEU466 PRO467 PHE468 GLY469 SER470 CYS475 ILE476 GLN512 THR513 GLY514 PHE515 ASN516
ent-kaur-16-en-19-oic acid	-8.2	LYS49 ALA52 ILE53 GLU59 HIS60 PHE61 TYR64 PHE83 HIS110 GLY111 PHE113 LEU114 TRP122 PHE127 SER128 ALA231 VAL232 ILE234 ALA239 ALA240 SER242 SER243 THR259 MET260 VAL333 ALA334 THR338 PRO399 THR400 VAL401 THR402 ALA403 GLU404 GLN512 PHE515 ASN516
[6]-gingerol	-6.2	LYS49 ALA52 ILE53 LYS58 GLU59 HIS60 PHE61 TYR64 ASN82 PHE83 HIS110 GLY111 SER112 PHE113 LEU114 TRP122 PHE127 SER128 ILE234 SER236 ALA239 ALA240 SER242 SER243 LEU246 THR259 VAL333 THR400 VAL401 THR402 ALA403 GLU404 ARG405 PHE428 ILE511 GLN512 THR513 GLY514 PHE515 ASN516

[6]-shogoal	-6.6	LYS49 GLU59 HIS60 PHE61 TYR64 PHE83 HIS110 GLY111 SER112 PHE113 LEU114 GLU119 TRP122 PHE127 SER128 PHE145 ILE205 ALA209 ALA231 ILE234 SER236 ALA239 ALA240 SER242 SER243 LEU246 THR259 MET260 ILE289 PHE331 VAL333 ALA334 GLY335 THR338 THR339 PRO399 THR400 VAL401 THR402 ALA403 GLU404 ARG405 PRO467 PHE468 GLY469 GLN473 SER474 CYS475 ILE476 GLY477 PHE480 ALA481 GLN512 THR513 GLY514 PHE515 ASN516
Cedrol	-7.2	LYS49 ALA52 ILE53 LYS58 GLU59 HIS60 PHE61 TYR64 PHE83 PHE113 ILE234 ALA239 ALA240 SER243 LEU246 THR400 VAL401 THR402 GLN512 THR513 GLY514 PHE515 ASN516
a-cadinol	-6.6	LYS49 ALA52 ILE53 LYS58 GLU59 HIS60 PHE61 TYR64 ASN82 PHE83 HIS110 PHE113 LEU114 TRP122 PHE127 SER128 ILE234 ALA239 ALA240 SER242 SER243 LEU246 VAL333 ALA334 THR400 THR402 ALA403 GLU404 GLN512 THR513 GLY514 PHE515 ASN516
Genistein	-7.6	PHE61 HIS110 GLY111 SER112 PHE113 LEU114 ASN116 TRP122 LEU126 PHE127 SER128 TRP134 ARG138 ALA334 GLY335 THR338 THR339 LEU342 LEU394 PRO398 PRO399 THR400 VAL401 THR402 ALA403 GLU404 ARG405 PRO467 PHE468 GLY469 GLN473 SER474 CYS475 ILE476 ALA481 SER485 PHE515 ASN516 MET517
Biochanin A	-8	PHE61 HIS110 GLY111 SER112 PHE113 LEU114 ASN116 GLU119 TRP122 PHE127 SER128 VAL232 ILE234 SER242 THR259 MET260 VAL333 ALA334 ASP337 THR338 THR339 PRO399 THR400 THR402 ALA403 GLU404 ARG405 PRO467 PHE468 GLY469 SER470 GLN473 SER474 CYS475 ALA481 PHE515 ASN516
Apigenin	-7	ASN57 HIS60 PRO62 ASP63 LEU66 ARG67 ARG70 HIS348 GLN349 VAL352 TYR353 MET396 PHE397 TYR432 HIS436 GLU445 ARG446 PHE447 LEU506 PRO507 MET508 LYS509 ILE510 ILE511 GLN512 GLU520
Glyceollin	-9.1	LYS49 ALA52 ILE53 GLU59 HIS60 PHE61 ASN82 PHE83 HIS110 GLY111 SER112 PHE113 LEU114 TRP122 PHE127 SER128 ALA231 VAL232 ILE234 SER236 ALA239 ALA240 SER242 SER243 LEU246 THR259 MET260 ALA330 VAL333 ALA334 GLY335 ASP337 THR338 PRO399 THR400 VAL401 THR402 ALA403 GLU404 ARG405 VAL431 LEU466 PRO467 PHE468 GLY469 GLN473 SER474 CYS475 GLN512 THR513 GLY514 PHE515 ASN516
α-gurjunene	-7.1	LYS49 ALA52 ILE53 LYS58 GLU59 HIS60 PHE61 TYR64 PHE83 PHE113 LEU114 TRP122 VAL232 ILE234 ALA239 SER242 SER243 LEU246 THR259 VAL333 PRO399 THR400 VAL401 THR402 ALA403 GLN512 THR513 GLY514 PHE515 ASN516

Alloaromadendrene	-7.5	LYS49 ALA52 ILE53 LYS58 GLU59 HIS60 PHE61 TYR64 PHE83 HIS110 PHE113 LEU114 TRP122 PHE127 ILE234 ALA239 SER242 SER243 THR259 VAL333 ALA334 THR400 THR402 ALA403 GLU404 LEU466 PRO467 PHE468 GLY469 GLN473 SER474 CYS475 GLN512 GLY514 PHE515 ASN516
Humulene	-7.2	PHE46 LYS49 ALA52 ILE53 LYS58 GLU59 HIS60 PHE61 TYR64 PHE83 PHE113 LEU114 TRP122 PHE127 ILE234 ALA239 SER242 SER243 THR259 VAL333 THR400 THR402 GLN512 THR513 GLY514 PHE515 ASN516
Caryophyllene	-7.5	LYS49 ALA52 ILE53 LYS58 GLU59 HIS60 PHE61 TYR64 PHE83 HIS110 PHE113 LEU114 TRP122 PHE127 ILE234 ALA239 SER242 SER243 THR259 ALA330 VAL333 ALA334 THR338 PRO399 THR400 VAL401 THR402 ALA403 GLU404 CYS475 GLN512 THR513 GLY514 PHE515 ASN516
Ferruginol	-8	PHE61 HIS110 GLY111 SER112 PHE113 LEU114 GLU119 TRP122 PHE127 SER128 ALA231 VAL232 LYS233 ILE234 SER242 THR259 MET260 VAL333 ALA334 ASP337 THR338 PRO399 THR400 VAL401 THR402 ALA403 GLU404 ARG405 VAL431 LEU466 PRO467 PHE468 GLY469 SER470 GLN473 CYS475 PHE515 ASN516
Manool	-7.7	LYS49 ALA52 ILE53 GLU59 HIS60 PHE61 PRO62 TYR64 PHE83 HIS110 GLY111 SER112 PHE113 LEU114 ASN116 TRP122 SER125 PHE127 SER128 ALA231 VAL232 ILE234 SER236 ALA239 ALA240 SER242 SER243 THR259 MET260 ALA330 VAL333 ALA334 THR338 THR400 THR402 ALA403 GLU404 CYS475 GLN512 THR513 GLY514 PHE515 ASN516
Nezukol	-7.8	PHE61 HIS110 GLY111 PHE113 LEU114 TRP122 PHE127 SER128 ALA231 VAL232 ILE234 SER242 THR259 MET260 ALA330 VAL333 ALA334 THR338 THR400 VAL401 THR402 ALA403 GLU404 PHE515 ASN516
Chamaecynone	-7.2	LYS49 ALA52 ILE53 LYS58 GLU59 HIS60 PHE61 TYR64 ASN82 PHE83 PHE113 LEU114 ILE234 ALA239 ALA240 SER242 SER243 LEU246 VAL333 THR400 VAL401 THR402 GLU404 PHE428 GLN512 THR513 GLY514 PHE515 ASN516
7-methyljuglone	-7.1	LYS49 ALA52 ILE53 LYS58 GLU59 HIS60 PHE61 TYR64 ASN82 PHE83 PHE113 LEU114 TRP122 ILE234 ALA239 SER242 SER243 LEU246 PRO399 THR400 VAL401 THR402 GLU404 ILE511 GLN512 THR513 GLY514 PHE515 ASN516

Citral	-5.5	LYS49 ALA52 ILE53 GLU59 HIS60 PHE61 TYR64 PHE83 HIS110 GLY111 SER112 PHE113 LEU114 ASN116 TRP122 PHE127 SER128 ALA231 ILE234 ALA239 ALA240 SER242 SER243 THR259 MET260 THR400 VAL401 THR402 ALA403 GLU404 PHE428 GLN512 GLY514 PHE515 ASN516
Citronellal	-5.9	PHE61 PHE113 LEU114 GLU119 TRP122 PHE127 PHE145 VAL204 ILE205 ALA208 ALA209 ARG230 ALA231 LYS233 ILE234 ALA239 SER242 THR259 MET260 ILE289 PHE331 VAL333 ALA334 GLY335 THR338 THR339 PRO399 THR400 VAL401 THR402 CYS475 ILE476 GLY477 PHE480 ALA481 GLY514 PHE515 ASN516
Eugenol	-6	PHE61 PRO62 HIS110 GLY111 SER112 PHE113 LEU114 ASN116 TRP122 PHE127 SER128 PHE145 ILE205 ALA209 ILE234 ALA239 SER242 ILE289 PHE331 ALA334 GLY335 THR338 THR339 LEU394 PRO398 PRO399 THR400 VAL401 THR402 ALA403 PRO467 PHE468 CYS475 ILE476 GLY477 PHE480 ALA481 ILE511 THR513 GLY514 PHE515 ASN516
Geraniol	-5.4	LYS49 ALA52 ILE53 LYS58 GLU59 HIS60 PHE61 TYR64 PHE83 GLY111 SER112 PHE113 LEU114 TRP122 PHE127 SER128 ILE234 ALA239 SER242 SER243 THR259 PRO399 THR400 VAL401 THR402 ALA403 GLU404 GLN512 THR513 GLY514 PHE515 ASN516 MET517
Limonene	-4.5	PHE61 PHE113 LEU114 GLU119 TRP122 PHE127 PHE145 ILE205 ALA208 ALA209 ALA231 VAL232 ILE234 ALA239 SER242 THR259 MET260 ILE289 PHE331 VAL333 ALA334 GLY335 PRO399 THR400 VAL401 THR402 CYS475 ILE476 GLY477 PHE480 ALA481 GLY514 PHE515 ASN516
Piperitone	-5.6	LYS49 ALA52 ILE53 LYS58 GLU59 HIS60 PHE61 TYR64 PHE83 PHE113 LEU114 TRP122 ILE234 ALA239 SER242 SER243 THR259 THR402 GLN512 THR513 GLY514 PHE515 ASN516
